# Clinicopathological spectrum and outcome of appendicectomy at a South African tertiary hospital: continuing impact of delayed presentation

**DOI:** 10.4314/ahs.v24i2.37

**Published:** 2024-06

**Authors:** PB Simelane, SS Kader, TE Madiba

**Affiliations:** Department of Surgery, University of KwaZulu-Natal, Durban

**Keywords:** Appendicitis, complicated appendicitis, treatment outcomes

## Abstract

**Introduction:**

Appendicitis is a common surgical emergency, associated with significant morbidity and mortality.

**Aim:**

To describe the clinicopathological spectrum and outcome of appendicitis in our setting.

**Methods:**

Retrospective descriptive chart review of patients undergoing surgery for appendicitis over two study periods (Group A: 2010-2012 and Group B: 2016-2018). Data collected included demographics, clinical presentation, operative findings and outcome.

**Results:**

There were 229 patients in Group A [median age 24 (IQR 18-32) years] and 145 in Group B [median age 28 (IQR 20-36) years]. Median pre-hospital delay was 3 days for Group A 3 (IQR 2-4) and 1 day for Group B 1 (IQR 1-2). Complicated appendicitis occurred in 69 (30.1%) and 37 (25.5%) patients in Group A and B respectively. Post-operative complication rate was 18.3% and 8.3 % in Groups A and B respectively. Postoperative mortality in Group A was 3.5% and 2 1% for Group B. Delay in presentation was associated with increased complicated appendicitis.

**Conclusion:**

Complicated appendicitis was seen in one third of the patients in both groups. Delay in presentation persists in our setting and it is associated with complicated appendicitis, which carries an increased morbidity and mortality.

## Introduction

Appendicitis is the most common cause of an acute surgical abdomen^1^–^3^. Despite advances in diagnosis and treatment, it is still associated with significant morbidity and mortality^1^,^4^. The aetiology is obstruction of the appendicular lumen, which facilitates intraluminal pressure build-up^3^. The appendix becomes inflamed and swollen in its disease course as intestinal bacteria multiply in the lumen, recruiting white blood cells and forming pus^3^. With this accumulation of purulent material, the intraluminal pressure rises, leading to wall ischaemia, necrosis and eventual rupture^3^. Rupture can then either lead to a contained abscess or peritonitis which, in turn, causes sepsis and potentially death^3^. Acute appendicitis affects both men and women of all races^5^ and the incidence peaks in children and young adults^5^,^6^. The estimated lifetime risk of developing acute appendicitis is 7-8%^4^,^7^.

For decades acute appendicitis has been known as a disease of populations in high income countries (HICs), and the literature has consistently suggested that its incidence is lower among Africans in low- and middle- income countries (LMICs)^6^,^8^. Recent studies, however, have shown an increase in the diagnosis of this condition among African patients^6^,^9^,^10^. Studies from South Africa have indicated an increasing incidence of acute appendicitis^3^,^11^,^12^, with White patients having 10-20 times more common occurrence compared to African patients^3^. The incidence rate among White South Africans is comparable to that in HICs, whereas that among Black South Africans is significantly lower^3^.

Kong et al published extensively on acute appendicitis in South Africa between the years 2012-2015 and highlighted late presentation as a key issue with morbidity (Wound sepsis, Pneumonia, Renal failure etc.) and mortality^1^,^12^–^15^. We hypothesized that the disease spectrum must have changed over time. We therefore undertook to study on the clinicopathological spectrum of acute appendicitis at King Edward VIII Hospital (KEH) covering two defined 3-year periods. The two periods were conveniently chosen and were three years apart. The breakdown in the filing system at KEH during the years 2012-2015 gave the authors to use this as an intervening period between the two three-year periods. The study sought to establish if trends had remains remained the same or had changed between the two periods. The study was mostly focused at auditing the influence of delay in presentation on intraoperatve findings and the influence of disease severity on outcome of management.

## Methods

The study was conducted at King Edward VIII Hospital (KEH), Durban. Durban is situated in the Eastern seaboard of the KwaZulu-Natal (KZN) Province of South Africa. Durban has six referral hospitals affiliated to the University of KwaZulu-Natal Medical School. KEH is one of the hospitals where appendicectomy can be undertaken. The objective was to audit all cases of surgically proven acute appendicitis over the 2 selected time periods and assess for morbidity and mortalities at KEH. The study included all patients who were found to have acute appendicitis at surgery for an acute abdomen. Exclusion criteria were: (i) patients who were managed non-operatively for acute abdomen as we wanted to review the surgical outcomes and complications only and of those patients who had surgical acute appendicitis, (ii) patients who were found to have peritonitis from other causes at laparotomy, (iii) patients in whom a provisional diagnosis of acute appendicitis was considered preoperatively but excluded at surgery.

This was a retrospective chart analysis of patients who underwent surgery for acute appendicitis managed at KEH. The first group of patients was over a three-year period from 1st January 2010 to 31st December 2012 (Group A) and the second group was from 1st January 2016 to 31st December 2018 (Group B). The details of patients with a diagnosis of acute appendicitis at operation were identified in the theatre operating books at KEH. The clinical information was then obtained from the clinical files collected from the Hospital Medical Registry at KEH. Data collected were entered onto a pre-designed data collection document and captured into an MsExcel computer database. Acute appendicitis was classified into simple appendicitis and complicated appendicitis. Simple appendicitis refers to an inflamed appendix without any pus collection or gangrenous changes. Complicated appendicitis occurred in patients with complications of appendicitis namely, perforation with localised appendix abscess or generalized peritonitis, gangrenous appendicitis, appendicular empyema (Obstructed canalization of the organ from purulent peritonitis) and mucocoele (Dilation of the appendiceal lumen as a result of mucin accumulation). The study outcome measures were morbidity, mortality and hospital length of stay. Patients were also stratified according to early presenters (≤1 day) and late presentation (> 1 day) depending on the time lapse from the first symptom to presentation at hospital. Pre-hospital is defined as the period prior to admission to the treatment hospital, hence primary health care and time spent at home with symptoms encompasses this term. In-hospital delay is defined as the period a patient is admitted to the definitive surgical hospital and is awaiting surgical intervention. Complications are graded according to Clavien Dindo 3 grading system. The manuscript was prepared according to the STROBE checklist.

The data were kept in a password-protected Microsoft Excel Spreadsheet. Stata V15.1 statistical software was used for the analysis. Measurements of central tendency were expressed as median and the interquartile range (IQR) which is a measure of statistical dispersion as the difference between the 75th and 25th percentiles of the data and enhances the accuracy of dataset statistics by dropping lower contribution, outlying points. For subgroup comparisons (Early presentation and late presentation subgroups of Group A and B) between categorical independent variables, the Chi-Square test was used to identify significant differences in frequencies of categorical variables by group, where numbers were very small, Fisher's exact test was used. Two sample Mann Whitney tests were used to compare medians test was performed to identify any significant differences in the mean rank of a given continuous explanatory variable by group (Group A vs Group B or male vs female) as the assumptions of the standard t-test were not met. A p-value of <0.05 was regarded as statistically significant.

Ethical approval for the study was obtained from the Biomedical Research Ethics Committee of the University of KwaZulu-Natal (Ref.: BE082/14), and the KwaZulu-Natal Department of Health (Ref.: 1178404).

## Results

**Figure 1:**
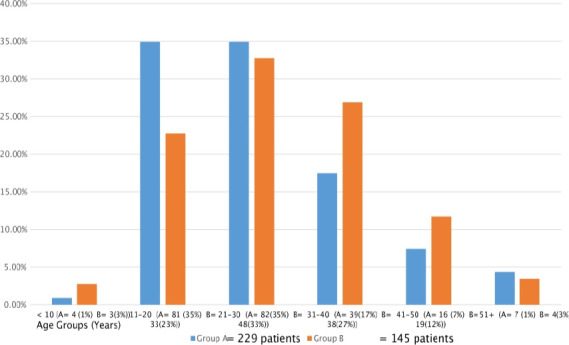


The patients were divided into 2 groups based on the above time intervals. For the first time period 241 files in Group A of which 12 had missing data leaving 229 files for analysis. The second time period, 154 patient files were found in Group B of which nine had missing data, leaving 145 files for analysis. The derivation of the study sample is shown in Figure 1 and patient profile of both groups is shown in Table I. Median age for Group A was 24 (IQR 18-32) years and that for Group B was 28 years (IQR 20-36). As can be seen in Figure 2, the peak age for Group A was in the second and third decade and the peak age for Group B was in the third decade. There were 142 males (62%) in Group A giving a male to female ratio of 1.6:1, whereas Group B comprised 80 males (55%) giving a male to female ratio of 1.2:1. Median pre-hospital delay was 3 (IQR 2-4) days for Group A and one day (IQR 1-2) for Group B. Two hundred and two patients in Group A (88%) and 54 patients in Group B (37%) had a delay of more than one day before presentation. Median in-hospital delay was 3 (IQR 2-4) hours for Group A and 2 hours (IQR 1-3) for Group B. Fever (182 (79.5%)), vomiting (176 (76.5%)) and right iliac fossa pain (163 (71.2%)) were the most common presenting symptoms in Group A. Classical pain, which commences in the umbilical region and migrates to the right iliac fossa, was the most common finding in Group B but only occurred in 11% in Group A. The routine blood results (Haemoglobin, White cell count, Platelets and Urea) were similar in both groups.

**Table I:** Demographics And Clinical Presentation In Patients With Appendicitis

Demographics		
*Description*	*Group A* *(n=229)*	*Group B* *(n=145)*
Age (years) median IQR	24 (IQR 18-32)	28 (IQR 20-36)
Males	142 (62.0%)	80 (55.2%)
M:F ratio	1.6:1	1.2:1
**Delay before presentation**		
***Delay***	***Group A*** ***(n=229)***	***Group B*** ***(n=145)***
Delay before presentation (< 1 day)	27 (11.8%)	91 (62.8%)
Delay before presentation (> 1 day)	202 (88.2%)	54 (37.2%)
Pre-hospital delay (days)	3 (IQR 2-4)	1 (IQR 1-2)
In-hospital delay (hours)	3 (IQR 2-4)	2 (IQR 2-3)
**Clinical Presentation**		
***Clinical feature***	***Group A*** ***(n=229)***	***Group B*** ***(n=145)***
Fever	182 (79.5%)	35 (24.1%)
Vomiting	176 (76.5%)	63 (43.5%)
Right iliac fossa pain	163 (71.2%)	49 (33.8%)
Clinical peritonitis	51 (22.3%)	29 (20%)
Non-specific abdominal pain	21 (9.2%)	14 (9.7°%)
Classical pain	26 (11.4%)	61 (42.1%)
Clinical appendix mass	12 (5.2%)	1 (0.7%)
Clinical appendix abscess	10 (4.4%)	0%
***Laboratory***		
Haemoglobin [Median (IQR)]	12.3 (11.2–13.5)	12.3 (11.5–13)
White cell count [Median (IQR)]	12.3 (9.9-14.2)	11.3 (9.6–13.8)
Platelets [Median (IQR)]	276(216-329)	233 (190-292)
Urea [Median (IQR)]	7.4 (5.8-9.2)	4.1 (3.3-5.2)

Numbers expressed in Median (IQR)

Clinical features expressed in numbers (%)

One hundred and sixty-three patients (71%) in Group A underwent appendicectomy via a Lanz incision. Of the 66 patients (28.8%) who underwent laparotomy, 59 (25.7%) had appendicectomy and seven had right hemicolectomy for an associated caecal perforation. In Group B, 106 patients (73.1%) underwent appendicectomy via a Lanz incision. Of the 39 patients (26.8%) who underwent laparotomy, 37 (25.5%) had appendicectomy and two had right hemicolectomy (1.3%) for an associated caecal perforation. Operative findings are shown in Table II. One hundred and sixty patients (69.9%) in Group A and 108 patients (74.5%) in Group B had an inflamed appendix, which turned out to be simple appendicitis. The rest had peritonitis and abscess collection both due to complicated appendix. Complicated appendicitis therefore was seen in 69 patients (31.1%) in Group A and 37 (25.5%) in Group B. Perforation was a major contributor to complicated appendicitis in both Group A and B where it accounted for 73.9% and 91.8% respectively.

**Table II:** Intraoperative and pathological findings in patients with appendicitis

Intraoperative findings		
*Finding*	*Group A* *(n=229)*	*Group B* *(n=145)*
Inflamed appendix	160 (69.9%)	108 (74.5%)
Peritonitis	56 (24.5%)	37 (25.5%)
Appendix abscess	3 (1.3%)	0%
**Pathological findings**		
***Finding***	***Group A*** ***(n=229)***	***Group B*** ***(n=145)***
Simple appendicitis	160 (69.9%)	108 (74.5%)
Complicated appendicitis	68 (30.1%)	38 (25.5%)
Perforated appendix	51 (73.9%)	34 (91.8%)
Gangrenous appendix	9 (13%)	1 (2.9%)
Perforated caecum	7 (10.1%)	2 (5.8%)
Empyaema	1 (1.4%)	1 (2.9%)

1 Perforation of both appendix and caecum

In Group A, nine patients (3.9%) required critical care admission postoperatively with median ICU stay of 5 days (IQR 3-6) and, in Group B, seven patients (4.8%) required critical care admission with a median ICU stay of 8 days (IQR 6-15). Eight patients (3.5%) in Group A and three patients in Group B (2.1%) died, all from multiple organ dysfunction syndrome (MODS). Table III shows postoperative complications stratified according to the Clavien-Dindo 3 classification of post-surgical complications (16). Post-operative complication rate was 18.3% and 8.3 % in Groups A and B respectively, of which the vast majority were seen in 41 patients (11%) within the delayed presentation subgroup being surgical site infection (20; 5.3%), prolonged ileus (8; 2.1%), hemorrhage (1; 0.2%), acidosis (5; 1.3%), MODS (5; 1.3%) and stump blow-out (1; 0.2%). Ten patients (4.4%) in Group A and 15 (10.3%) in Group B required planned relook laparotomy.

**Table III:** Complications in patients undergoing surgery for appendicitis stratified according to the clavien-dindo version 3 classification

Presentation		Early presentation		Delayed presentation	
*Clavien-Dindo Classification*	*Complication*	*Group A* *n=229*	*Group B* *n=145*	*Group A* *n=229*	*Group B* *n=145*
Total	Patients with complications	0	1 (0.7%)	29 (12.7%)	12 (8.3%)
Grade I	Surgical site infection	0	1 (0.7%)	13 (5.6%)	7 (4.8%)
	Prolonged ileus	0	0	3 (1.3%)	5 (3.4%)
	***Sub-total***	***0***	***1(0.7%)***	***14 (6.1%)***	***2 (1.4%)***
Grade II		0	0	0	0
					
Grade III	Peritonitis	0	0	3 (1.3%)	1 (0.7%)
	Hemorrhage	0	0	1 (0.4%)	0
	Acidosis	0	0	3 (1.3%)	2 (1.3%)
	***Sub-total***			***4 (1.8%)***	***1 (0.7%)***
Grade IV	MODS	0	0	1 (0.4%)	4 (2.7%)
	Peritonitis	0	0	0	7 (4.8%)
	Stump blow-out	0	0	1 (0.4%)	0
	***Sub-total***			***1 (0.4%)***	***7 (4.8%)***
Grade V (Mortalities)	Renal dysfunction	0	0	6 (2.6%)	3 (2%)
	MODS	0	0	8 (3.4%)	3 (2%)
	Peritonitis	0	0	8 (3.4%)	3 (2%)
	Acidosis	0	0	8 (3.4%)	3 (2%)
	***Sub-total***	***0***	***0***	***8 (3.5%)***	***3 (2.1%)***

The subtotal and total are the numbers of patients with the complications and not the number of complications

Table IV shows the influence of delay in presentation on outcome. In Group A there was a significant association between late presentation and complicated appendicitis. Morbidity (p=0.001) and mortality (p=0.60) were more common in patients with delayed presentation. Critical care admission was significantly more common in patients with delayed presentation. Delay in presentation had no influence on hospital stay in Group A (p=0.2). In Group B, delay in presentation was associated with increased morbidity, and critical care admission as well as longer hospital stay. There was no significant difference in mortality (p=0.5).

**Table IV:** Influence of delay in presentation on outcome

Group A			
Delay in intervention	< 1 day n=27 (11.8%)	> 1 day n=202 (88.2%)	p-value
Complicated appendix [n (%)]	2 (7.4%)	67 (33.2%)	0.006 ^3^
ICU admission [n (%)]	0	9 (4.5%)	0.61^1^
Mortality [n (%)]	0	8 (4%)	0.60^1^
Postoperative complications [n (%)]	2 (7.4%)	40 (19.8%)	0.18^1^
Hospital stay [days, median IQR]	4.0 (3-6)	4.0 (3-6)	0.2^2^
**Group B**			
**Delay in intervention**	**< 1 day** **n=91 (62.8%)**	**> 1 day** **n=54 (37.2%)**	**p-value**
Complicated appendix [n (%)]	1 (1.1%)	36 (66.7%)	<0.001 ^3^
ICU admission [n (%)]	0	7 (13.0%)	<0.001 ^1^
Mortality [n (%)]	0	3 (5.6%)	0.05 ^1^
Postoperative complications [n (%)]	0	12 (22.2%)	<0.001^1^
Hospital stay [days (median, IQR)]	4(3-7)	8.5 (6-15)	<0.001^2^

Legend

1 – Fisher's Exact Test

2 – Mann-Whitney Test

3 – Chi squared Test

## Discussion

This study sought to quantify outcome of surgical management of acute appendicitis with special reference to delay in presentation and complexity of disease. Emphasis was on two research periods, three years apart. The median age of 24 and 28 years for Group A and B respectively was in keeping with the world literature, which reports the age of presentation at 29-42 years^2^,^17^. There are many reasons for the smaller number of patients in Group B. The intrinsic referral pattern may have changed, patients could have gone to alternative hospitals within the city of Durban, KEH was closed for a short time in the intervening years between the first period and second period. A male preponderance was observed in both groups. The dominance by a male gender is reported international studies^2^,^7^,^18^,^19^, and is also seen in other African Studies^3^,^11^,^12^,^14^,^20^, although a few studies in Africa report a female preponderance^5^,^9^. Interestingly, a study by Kong et al conducted in Pietermaritzburg, South Africa, observed male preponderance in urban patients with acute appendicitis but a female preponderance in rural patients with acute appendicitis^13^.

The main observation is that delay was a major factor in both patient groups, which in turn predisposed to complicated appendicitis, both of which led to increased morbidity and mortality as well as prolonged hospital stay. The median pre-hospital delay of three days in the first period improved to one day in the second period. The proportion of patients presenting late was much higher in Group A than in Group B.

Complicated appendicitis was seen in about a third of cases in both groups in this series. In a study in Pietermaritzburg, KZN, Kong et al reported a rate of complicated appendicitis to be 57%^1^. When the same authors stratified their patients into rural and urban, they found a complicated appendicitis rate of 79% in rural women, whereas in urban women, complicated appendicitis was seen in 48% of cases^12^. The perforation rate was 25.3% and 24.8% both of which falls within the 22-60% from other studies from sub-Saharan Africa^1^,^13^, but far exceeds the rates of 14-30% seen elsewhere in the international literature^1^,^13^. This shows that the perforation rate did not change over the two periods. The postoperative complication rate was 12.3% and 8.3% in Group A and B respectively and there were significantly more complications among patients with complicated appendicitis in both groups. The mortality rate of 3.1% in the first period was quite high compared to the 0.28-2% reported in the literature^2^,^3^,^11^,^13^,^21^,^22^, but the mortality rate in the second period fell within this range.

The conventional pathophysiologic model of acute appendicitis is based on a relationship between time and disease progression; hence, the risk of perforation increases as time elapses from onset of disease to treatment^17^,^23^. Since intra-abdominal sepsis is a time-dependent condition, delayed surgical source control is directly associated with adverse outcomes^15^,^24^,^25^. More severe presentations of complicated appendicitis are associated with worse outcomes and greater resource use including significant postoperative morbidity, readmissions, length of stay and mean cumulative cost^14^,^19^,^24^. Drake et al, on the other hand, concluded that perforation in acute appendicitis was not associated with lapsed time from hospital admission to commencement of opertion of appendicectomy but rather a prehospital event^17^. Drake et al further concede that perforation may not be strictly a time-dependent phenomenon and that other factors may be at play^17^. Although the extent of delay is lower in the second compared to the first period, the study has demonstrated no change in the incidence of complicated appendicitis and prognosis of appendicitis in our setting. Clinical and biochemical features suggestive of complicated appendicitis include fever, vomiting, longer duration of symptoms, elevated CRP level or WBC count, and ultrasound findings of free abdominal fluid, visualized perforation, or a mean appendix diameter of 11 mm or more^26^.

In high income countries (HIC's), early recognition and timely surgical therapy have dramatically reduced the morbidity and mortality related to acute appendicitis^22^. Unfortunately, acute appendicitis continues to have disparate outcomes in different populations around the world and remains associated with increased severity and worse outcomes in South Africa when compared to acute appendicitis in other LMICs^22^,^27^,^28^. Several other South African studies have shown that patients with acute appendicitis experience significant delays between the onset of symptoms and definitive surgical treatment, resulting in significant morbidity and poorer outcomes as well as substantial cost to the healthcare system^3^,^13^,^15^. We share the view expressed by others^15^ that improvement to the diagnostic capability of healthcare workers in developing countries need to be addressed as a matter of urgency.

Although the number of patients with delay of greater than one day was smaller in Group B, delay in presentation remains a problem in our setting, suggesting that a need exists for more efforts in addressing delay in presentation. Attempting to reduce postoperative complications in patients undergoing appendicectomy especially following surgery for complicated appendicitis is no easy task. These include intraoperative risk stratification to determine optimal postoperative management^29^, the use of wound edge protectors to reduce surgical site infection^29^ and the use of prophylactic antibiotics for simple appendicitis and therapeutic antibiotics for complicated appendicitis^29^. Attempts to reduce delay before presentation will depend mainly on the population's understanding of the disease, its symptoms, and complications. Likewise, healthcare workers at the lower-level facilities need to understand the disease and the implications of delay and, as such, they should be empowered with clinical prediction rules or guidelines for acute appendicitis; and widespread awareness campaigns aimed at vulnerable populations should be encouraged, by for example placing teaching materials in the general information health website for the South African population.

The study does have some limitations. It was a retrospective study, and the only inclusion criterion was a finding of acute appendicitis at surgery as opposed of preoperative diagnosis. The grouping was based on historical comparison and was not randomized. It was difficult to establish the precise time of symptom onset and as such, this information depended on the patients' history. The grouping of the two groups is based solely on historical data between the two groups. Also, since several hospitals within the city of Durban can undertake appendicectomy, patients have a large choice, and it is easy for them to move between hospitals. The confounding factor, however, is that other hospitals can do appendicectomy and, in times of accommodation constraints, patients move between hospitals. The strength of the study is that it quantifies the differences in outcome between simple and complicated appendicitis and attempts to increase our understanding of the association between clinical presentation, intraoperative findings, and outcome. The study also involves two study periods, which attempts to establish changes in trends between the two time periods.

## Conclusion

Delay in the presentation of patients with acute appendicitis to hospital continues to be a major problem in our setting. The study provides an insight into the persistence of complicated appendicitis in our setting despite previous studies drawing attention to this entity. Complicated appendicitis was seen in one third of the patients in both groups, despite improvement in delay before presentation between the two study periods. The high rate of complicated appendicitis persists with associated morbidity and mortality and resultant increase in length of hospital stay. Acute appendicitis remains a morbid condition in low and middle-income countries. Managing complications of acute appendicitis is costly and reduces quality of life in these patients.
